# Hunting down zinc(II)-binding sites in proteins with distance matrices

**DOI:** 10.1093/bioinformatics/btad653

**Published:** 2023-10-25

**Authors:** Vincenzo Laveglia, Milana Bazayeva, Claudia Andreini, Antonio Rosato

**Affiliations:** Department of Chemistry, University of Florence, Sesto Fiorentino 50019, Italy; Department of Chemistry, University of Florence, Sesto Fiorentino 50019, Italy; Magnetic Resonance Center (CERM), University of Florence, Sesto Fiorentino 50019, Italy; Department of Chemistry, University of Florence, Sesto Fiorentino 50019, Italy; Magnetic Resonance Center (CERM), University of Florence, Sesto Fiorentino 50019, Italy; Consorzio Interuniversitario di Risonanze Magnetiche di Metallo Proteine, Sesto Fiorentino 50019, Italy; Department of Chemistry, University of Florence, Sesto Fiorentino 50019, Italy; Magnetic Resonance Center (CERM), University of Florence, Sesto Fiorentino 50019, Italy; Consorzio Interuniversitario di Risonanze Magnetiche di Metallo Proteine, Sesto Fiorentino 50019, Italy

## Abstract

**Motivation:**

In recent years, high-throughput sequencing technologies have made available the genome sequences of a huge variety of organisms. However, the functional annotation of the encoded proteins often still relies on low-throughput and costly experimental studies. Bioinformatics approaches offer a promising alternative to accelerate this process. In this work, we focus on the binding of zinc(II) ions, which is needed for 5%–10% of any organism’s proteins to achieve their physiologically relevant form.

**Results:**

To implement a predictor of zinc(II)-binding sites in the 3D structures of proteins, we used a neural network, followed by a filter of the network output against the local structure of all known sites. The latter was implemented as a function comparing the distance matrices of the Cα and Cβ atoms of the sites. We called the resulting tool Master of Metals (MOM). The structural models for the entire proteome of an organism generated by AlphaFold can be used as input to our tool in order to achieve annotation at the whole organism level within a few hours. To demonstrate this, we applied MOM to the yeast proteome, obtaining a precision of about 76%, based on data for homologous proteins.

**Availability and implementation:**

Master of Metals has been implemented in Python and is available at https://github.com/cerm-cirmmp/Master-of-metals.

## Graphical Abstract

**Graphical Abstract:**
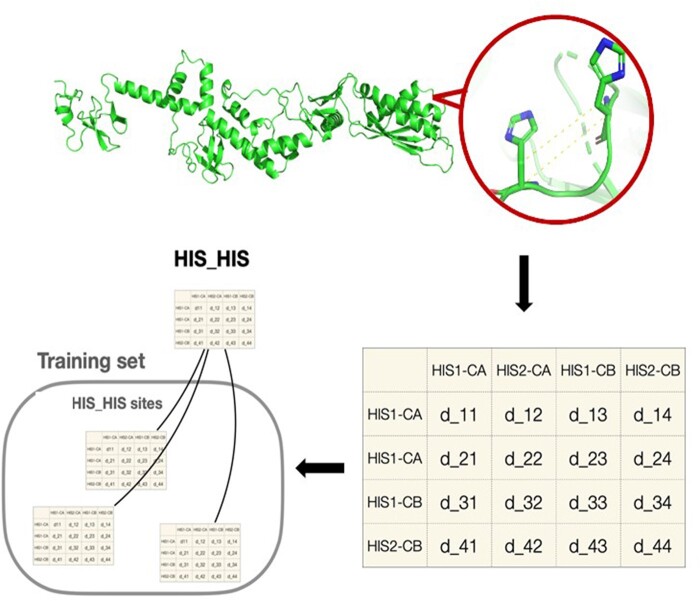

## 1 Introduction

Metalloproteins (MPs) are a diverse class of proteins that contain metal ions as integral components of their structures. They are found in all forms of life, from bacteria to humans, and are involved in numerous physiological processes, including catalysis, electron transfer, oxygen transport, and gene regulation. Metal ions can have a variety of roles in MPs. They can act as structural elements, providing stability to the protein, or they can participate in catalysis, activating substrates or stabilizing reaction intermediates. Metal ions can also act as electron carriers, transferring electrons between redox-active sites, or they can regulate protein activity and transduce cellular signals (Mertz 1998, Degtyarenko 2000, Nordberg and Nordberg 2016). MPs are also important targets for drug development. Many drugs target MPs by exploiting their metal-binding sites (MBSs) to block their activity (Chen *et al.* 2019). During bacterial infections, the host can deploy a protective mechanism, called “nutritional immunity,” which inhibits the growth of pathogens by restricting the availability of metal ions (Hennigar and McClung 2016).

The investigation of MPs at the whole organism or whole cell level is called metalloproteomics (Shi and Chance 2011, Barnett *et al.* 2012). Owing to the difficulties of experimental metalloproteomics, bioinformatics has rapidly emerged as an alternative approach to mine metalloproteomes (Andreini *et al.* 2009, Gladyshev and Zhang 2013, Zhang and Zheng 2020). In this context, the 3D structure-based prediction of the occurrence of metal sites, which makes use of the knowledge about the relative location in space of the amino acids possibly serving as donor atoms for metal coordination, is an area of application that has attracted a lot of attention (Andreini and Rosato 2022). The success of AlphaFold (Senior *et al.* 2020) and AlphaFold2 (Jumper *et al.* 2021a, b) in the CASP programs has given these kinds of approaches a considerable boost, thanks to the extensive availability of viable 3D structural models for proteins not yet described experimentally (Varadi *et al.* 2022).

There are several tools available to figure out a protein’s metal content [e.g. ZincFinder (Passerini *et al.* 2007), ZincExplorer (Chen *et al.* 2013), Zincbindpredict (Ireland and Martin 2021)], the residues that bind a metal [e.g. IonCom (Hu *et al.* 2016), MIB (Lin *et al.* 2016)], and the location of the metal [e.g. AlphaFill (Hekkelman *et al.* 2023), BioMetAll (Sánchez-Aparicio *et al.* 2021)]. These predictors use sequence and/or structural information as their input. Pattern recognition is used by sequence-based predictors to pinpoint the amino acids that could bind a metal. In structure-based approaches, the position of metals is inferred via distance characteristics (BioMetAll) or homology to known structures (MIB, AlphaFill). Some sequence-based approaches use machine learning (ML) techniques. Recently, a tool (Metal3D) exploiting 3D convolutional neural networks (NNs), a deep-learning methodology, became available to predict the location of zinc(II) ions in protein structures (Dürr *et al.* 2023).

In this work, we describe an approach for the prediction of zinc(II) MPs based on 3D structural models generated by AlphaFold that leverages a collection of metal site templates, i.e. a pre-arranged spatial distributions of prospective metal ligands. In our methodology, triads or quadruplets of amino acids with appropriate relative spatial arrangements are identified by a ML algorithm and then ranked based on their structural similarity to a library of templates extracted from the MetalPDB database (Andreini *et al.* 2013, Putignano *et al.* 2018). Our tool, called Master of Metals (MoM), can process an entire proteome in a few tens of minutes, with satisfactory accuracy. MoM is available at https://github.com/cerm-cirmmp/Master-of-metals.

## 2 Materials and methods

### 2.1 Representation of the metal-binding sites

Selecting informative features is the first step in the design of a successful ML/statistical model. In our context, several different features describing the chemical physical properties of MBSs could be used (Koohi-Moghadam *et al.* 2019, Feehan *et al.* 2021, Ireland and Martin 2021). In this work, we focused simply on the spatial configuration of the metal ligands. Thus, our input consisted of the Cartesian coordinates of the Cα and Cβ atoms, together with the amino acidic type. We decided not to use the coordinates of further atoms in the side chains (SCs), because we previously observed that metalation of an apo-MBS is likely to induce a significant rearrangement of the SCs, whereas the backbone atoms are largely unaffected (Bazayeva *et al.* 2023). The backbone atoms should thus be at a position that is closer to the holo-structure than the SC atoms in experimental or predicted apo-structures. A five-dimensional one-hot vector was used to specify the amino acidic type; the first 4 positions indicate if each residue is one among Cys/His/Asp/Glu [CHED group (Babor *et al.* 2008)], whereas the fifth was used for all other aminoacids. In this way, a *L*-length structure (excluding Gly) is represented by a 2*L* × 3 matrix containing the *x*, *y*, *z* coordinates of the Cα and Cβ atoms of each residue and a *L* × 5 matrix indicating the type of amino acid at each position.

### 2.2 Construction of the dataset of positives (holo sites and apo sites)

All zinc(II) sites annotated as “physiological,” and therefore having a functional role in the protein, were selected from MetalPDB (Andreini *et al.* 2013, Putignano *et al.* 2018). We retained only those having a single zinc(II) ion (mononuclear sites) with three or more protein residues acting as metal ligands. The MBSs were then grouped on the basis of their metal-binding pattern (i.e. the type and order of amino acids that bind directly to the metal ion). For example, all sites that bind the zinc(II) ion with a His followed in sequence by two Glu residues are grouped together as “His-Glu-Glu.” MetalPDB computed these patterns only within individual chains, thus making the current implementation of MoM not suitable to detect inter-chain sites. To avoid analyzing similar MBSs several times, we subsampled sites with the same pattern if there were more than 30 of them. For this, we computed the difference between all possible pairs of MBSs having a given pattern. Such difference is mathematically defined as the mean absolute value of the difference between the adjacency matrices of the two MBSs, where the adjacency matrix of an MBS is the matrix containing the distances among all the Cα and Cβ atoms of the MBS. For all MBSs having a difference smaller than a threshold *T* (set at 0.1 Å) only one site was retained. Note that the specific identity of the retained MBS is not relevant as MoM works only with the adjacency matrices. At the end of the subsampling, all remaining MBSs sharing the same pattern have a distance from each other greater then T. To remove protein redundancy due to homology, we clustered all the proteins with at least 30% identity in any given pattern-based group by running the CD-HIT (Huang *et al.* 2010) program. Five sites were randomly selected for each cluster and included in the dataset of holo (i.e. metal loaded) MBSs. Sites belonging to the same cluster were never separated into different datasets for algorithm training, i.e. were all included in the training dataset or all included in the testing dataset.

MetalPDB contains information also on apo (i.e. devoid of their physiological metal cofactor) MP structures (Putignano *et al.* 2018). Thus, we retrieved all apo-sites linked to mononuclear zinc(II) MBSs. We retained only one apo-site for each protein.

### 2.3 Construction of the dataset of negatives

To construct the dataset of negatives, i.e. non-zinc(II)-binding structures, we started from all sequences in the entire PDB and grouped them with CD-HIT (Huang *et al.* 2010) into clusters of sequences with at least 30% identity. All clusters containing one or more physiological metal-binding structures were then removed from the dataset. Finally, one structure from each remaining cluster was randomly selected.

### 2.4 MBS recognition pipeline/workflow

Our tool (MoM) takes as input a pdb or mmCIF file. For each CHED residue, MoM creates a group of CHED structural neighbors, whose Cα distances among each other are within a given threshold. In this way, we extract a list of potential sites (PSs) from the protein structure. The threshold values were defined from a previous analysis (Bazayeva *et al.* 2023) and taken equal to 13 Å. This procedure ensures that the residues in each PS are at reasonable distances, but we still do not know anything about their spatial configuration. We trained a graph neural network (GNN) to estimate the probability that a PS is an MBS (see next section). The PSs that have a probability value greater than .6 are named highly probable potential sites (HPPSs).

In practice, only some of the HPPSs are indeed real MBSs. To address this point, MoM compares each HPPS with all the MBSs of our training set that have the same metal-binding pattern (i.e. the type and order of amino acids that bind to the metal ion). For this comparison, all sites are represented as the adjacency matrices of their Cα and Cβ atoms. For each HPPS, MoM identifies the MBS having the smallest difference to it (*d*_min). If *d*_min is lower than a given threshold (e.g. 0.35 Å), we propose that the HPPS is a real MBS. Fundamentally, this is grounded on the fact that there exists an experimentally validated MBS that has a shape, as defined by the positions of the Cα and Cβ atoms, very similar to the predicted HPPS.

### 2.5 Architecture, training, and evaluation of performance of the GNN

MBSs can be represented suitably as graphs, where the Cα and Cβ atoms are the nodes, and the edges represent the interaction with neighboring atoms. GNNs are ML models engineered to process data structured as graphs. The nodes of the graph are associated to vectors that represent their state, i.e. their feature values. The topology of the graph, that is the set of relationships between all its nodes, is represented by the adjacency matrix *A*, whose (*i*, *j*)th element is 1 if node *i* and node *j* are connected by an edge and 0 otherwise. In our case, values in *A* are scaled as exp(−*d*_*ij*/alpha) where *d*_*ij* is the Euclidean distance between node *j* and node *i* and alpha is experimentally optimized.

In this work, we used a graph convolutional network (GCN). GCNs take as input the adjacency matrix and the feature vectors of the nodes. A GCN is composed by multiple stacked layers. Each layer generates a new feature vector (called embedding) for each node, processing its feature vector and those of the nodes to which it is connected. Lastly, the embeddings of all the nodes are averaged and the resulting vector is fed to a fully connected layer with two outputs, acting as the classifier.

To train the GCN, the dataset of holo sites was randomly split into validation (20%) and training (80%) groups. All the sites belonging to the same CD-HIT cluster (see Section 2.2) were assigned to the same group, thus ensuring that related MBSs are not found in different groups. The parameters of the model were optimized to maximize the performance of the training set (training process) using cross-validation and then tested on the validation set.

### 2.6 Evaluation of performance

In the holo and apo datasets, each item is a single MBS (target site); this means that we have multiple items for proteins harboring multiple MBSs. The negative dataset is composed of whole protein structures. We used the holo training set to optimize our GCN, which was then tested on the holo validation set, as well as on the apo and negative datasets. In our workflow, for the holo and apo datasets, a prediction is considered a success (true positive) if the site corresponds (at least two out of three metal ligands) to an MBS. Conversely, all experimental MBSs for which there was no prediction with a *d*_min value below the selected threshold are false negatives (FN). All predictions with a d_min value below the threshold for the structures in the negative dataset are false positives (FP). For our analyses, we used different performance measures, including:

Recall: TP/(TP + FN), also called sensitivity, true positive rate (TPR)

Precision: TP/(TP + FP), also called positive predictive value (PPV)

True negative rate (TNR): TN/(TN + FP)

False positive rate (FPR): FP/(TP + FP)

The same parameters were used to evaluate the results for the prediction of the zinc(II) proteome of yeast.

## 3 Results

The MBS is a substructure around the metal ion(s) that represents the macromolecular environment that the metal is sensing and can be automatically extracted from the 3D structures stored in the Protein Data Bank (PDB) (wwPDB Consortium 2019). This substructure ought to match the bare minimum environment that determines the functionality of the metal, or the “minimal functional site” (Andreini *et al.* 2011). In this work, we used the definition of MBS implemented in MetalPDB (Andreini *et al.* 2013, Putignano *et al.* 2018); alternative definitions tend to yield similar results (Tran and Krężel 2021). We implemented a ML approach to predict MBSs in the 3D structures of proteins, which we called MoM.

The PDB contains two different types of structures of MPs, depending on whether the deposited structure contains the physiological metal ion(s). In holo structures, the metal is present and thus the SCs of the metal-binding protein residues (the metal ligands) are organized such that their donor atoms have a spatial configuration matching the coordination geometry preferences of the metal (Andreini *et al.* 2012, Zheng *et al.* 2017). In apo-structures instead, the conformation of the SCs of the metal ligands may differ with respect to their counterparts in holo structures due to the absence of the metal ion. In particular, the donor atoms may be located at incorrect distances from one another and with the wrong geometry for metal binding (Bazayeva *et al.* 2023). We used only the holo structures to train MoM, whereas both apo and holo structures were employed to evaluate the tool performance.

### 3.1 Experimental datasets

To generate the holo structure dataset, we selected from MetalPDB (Andreini *et al.* 2013, Putignano *et al.* 2018) all the physiologically relevant zinc(II) MBSs (Laveglia *et al.* 2022) and retained only those having a single metal ion (mononuclear sites) with three or more donor residues. We first grouped the sites by their metal-binding patterns. The distribution of these patterns is very unbalanced, with the 12 most frequent patterns, covering 70% of all MBSs (Supplementary Table S1). We retained only the patterns observed in at least 10 sites.

For each pattern present in more than 30 MBSs, we subsampled all the sites to obtain a smaller dataset, more uniformly distributed in the MBS space. Subsequently, for each pattern, we clustered all the MP structures based on their sequence similarity. The sites belonging to the same cluster were all at once selected exclusively for inclusion in the training or test datasets, thus ensuring that the two datasets did not contain similar proteins. MetalPDB contains information also on apo-structures (Putignano *et al.* 2018), allowing us to retrieve all apo-sites linked to mononuclear zinc(II) MBSs. We kept only one apo-site for each MBS. In addition, to establish a negative dataset for validation, we chose an ensemble of proteins for which no metal containing structure was present in the PDB. In total, our datasets contained 3083 holo sites, 231 apo, sites and 500 negative proteins.

### 3.2 Performance of Master of Metals

For the holo and apo datasets, we consider a prediction to be correct if the known site is included among the sites output by MoM. Table 1 reports the recall obtained for different values of the *d*_min threshold (see Section 2), showing recall rates between 83% and 95% for holo MBSs. For the apo data, the recall ranges between 66% and 86%. This lower recall is determined by the structural rearrangements caused by metal binding in a protein site (Bazayeva *et al.* 2023). Indeed, our tool exploits the position of Cα and Cβ atoms precisely because their extent of rearrangement upon metalation is typically less extensive than that of SCs. This resulted in a still satisfactory recall of 66% for apo-structures at the most stringent *d*_min threshold, with a corresponding FPR of only about 7%.

**Table 1.** Performance of MOM.^a^

	Recall/TPR (%)		FPR (%)
*d*_min threshold	Holo data	Apo data	Negative data
0.25	82.7 ± 2.4	66.0 ± 1.4	6.60 ± 0.37
0.30	88.9 ± 1.2	74.3 ± 1.2	10.5 ± 0.2
0.35	91.9 ± 1.3	79.1 ± 1.6	14.2 ± 0.6
0.40	94.0 ± 1.3	80.6 ± 1.9	18.8 ± 0.5
0.45	95.2 ± 1.4	85.8 ± 1.9	25.0 ± 0.8

a We measured the performance as the fraction of correctly predicted sites over the total number of experimental sites, TP/(TP + FN). In addition, we used the structures of the negative dataset to estimate the FPR, given by the fraction of FP predictions over the total number of negative proteins in the dataset.

### 3.3 Structure-based prediction of zinc MBSs in the *Saccharomyces cerevisiae* proteome

To perform a proteome-wide prediction of zinc(II) MBSs in *S.cerevisiae*, we retrieved all the 6309 structural models of yeast proteins available from the AlphaFold database (Hekkelman *et al.* 2023). AlphaFold models include a measure indicative of the local accuracy of the prediction (plDDT). We retained the structures having at least 90% of their residues with a plDDT > 0.7, reducing the dataset to 1500 models, in order not to bias the prediction results due to the quality of the AlphaFold models. Within the latter ensemble, we identified 191 zinc(II)-binding proteins (Supplementary Table S2).

**Figure 1.**
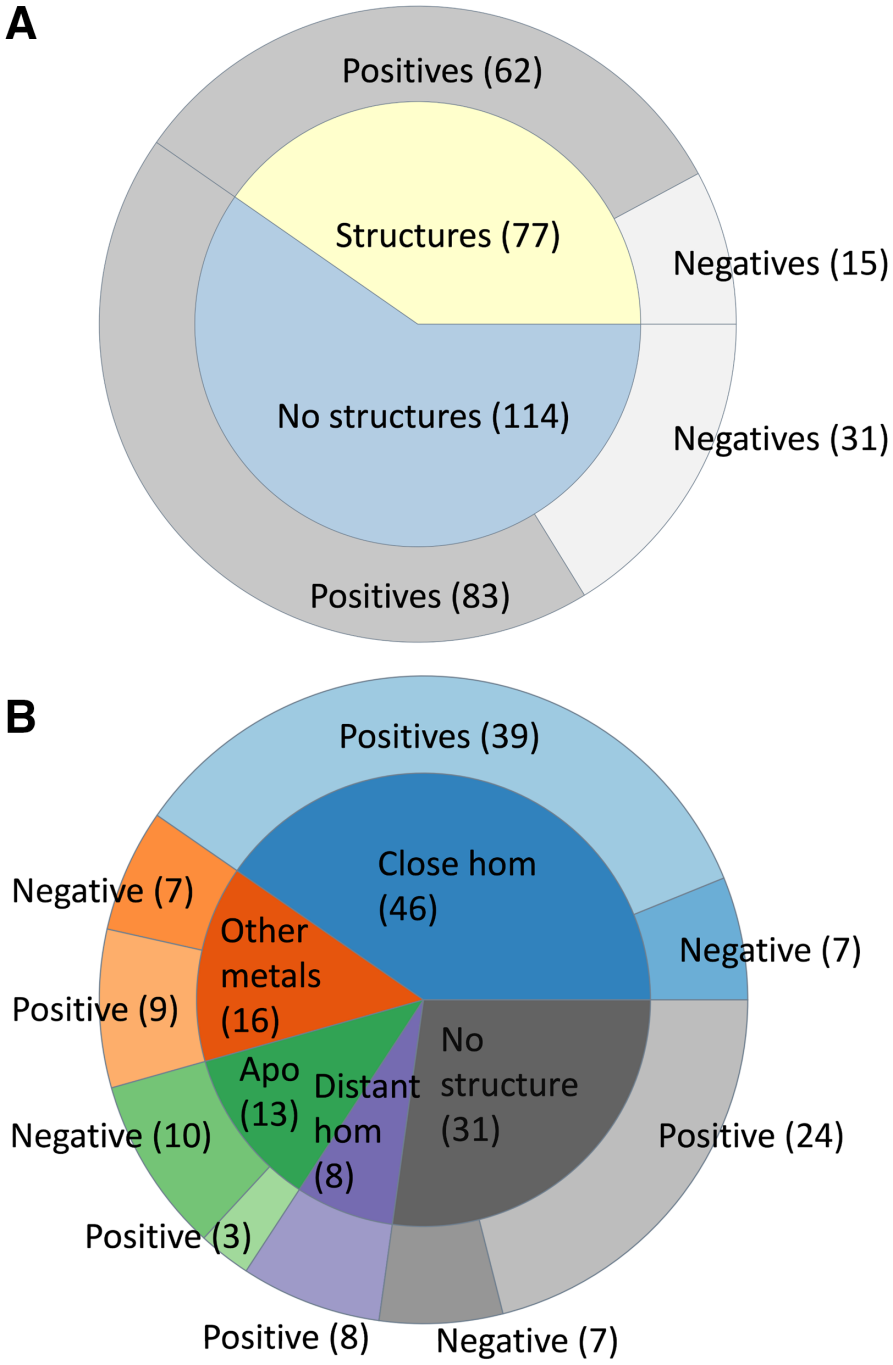
Validation of MOM against the *S.cerevisiae* proteome. (A) correct (dark gray, “positive”) and wrong (light gray, “negative”) predictions (outer doughnut) based on yeast proteins with deposited structure (77 proteins, inner doughnut, yellow), or on the structures of homologous proteins as well as visual inspection (114 proteins, inner doughnut, light blue); (B) breakdown of the validation based on the 114 proteins in the light blue wedge of panel (A), as a function of their characteristics (inner doughnut, compare to the columns of Table 2), showing correct and wrong predictions (outer doughnut; light and dark colors, respectively) for each group. The total number of positives in panel (A) is 145, whereas the total number of negatives is 46

For all the yeast proteins with a predicted MBS, we searched if there was already an experimental structure, by mapping their UniProt IDs to the PDB. Out of 191 proteins, 77 had a deposited structure and we observed that in 62 cases the MBS was correctly identified. This corresponds to a precision (PPV) of 80.5% and a false discovery rate of 19.5% (Fig. 1A).

We then looked for structurally characterized homologs of the remaining 114 proteins. BLAST retrieved close homologs having a deposited 3D structure for 75 proteins (Fig. 1B). Forty-six of these structures (61%) contain one or more zinc(II)-binding sites, 16 bear different metals than zinc(II) (21%), and 13 are apo structures (17%). For 8 proteins, only distant homologs were detected (i.e. BLAST retrieved some hits that did not fulfill our thresholds), whereas for 31 proteins no related structures were found. The latter group included nine proteins for which an experimental structure is available but lacks the region containing the predicted site because of the presence of an additional domain or motif in the AlphaFold model. For each yeast protein having a structurally characterized homolog, we superimposed its AlphaFold model to the experimental structure of the homolog. We assumed that if the homolog harbored an experimental zinc(II) MBS, then the predicted AlphaFold structure should also have a zinc(II) MBS.

For the 13 experimental apo structures, we qualitatively evaluated whether the spatial disposition of the residues in the predicted site suggested that it could be populated by a zinc(II) ion under appropriate conditions. In fact, it can happen that even in the 3D structure of an actual MP, the MBS is not populated by its cognate metal, because of shortcomings in the sample handling procedures (Grime *et al.* 2020). However, given that any incorrect prediction made by our tool can only be attributed to an apo structure, we anticipated that the predictions whose homologs are apo proteins would have the highest FP rate. Assuming mild rearrangements of the protein backbone, we determined that the predicted sites for 10 proteins in this group (76%) were unreliable because the disposition and/or orientation of the putative ligands was not appropriate for metal binding. However, three proteins (or 23% of all proteins in this group) had sites resembling physiological ones (see Table 2).

**Table 2.** Results of the inspection of the structural models of the 114 predicted zinc(II) proteins lacking an experimental structure.^a^

	Unreliable	Good/partial match	Perfect match	Total
Homologs with zinc	7 (15%)	7 (15%)	32 (70%)	46
Homologs with different metals	7 (44%)	4 (25%)	5 (31%)	16
Apo homologs	10 (77%)	3 (23%)	n.a.	13
Distant homologs	n.a.	2 (25%)	6 (75%)	8
No homologs or no corresponding region	7 (23%)	13 (42%)	11 (35%)	31
Total	32 (28%)	28 (24%)	54 (47%)	114

a Distant homologs are the proteins identified by BLAST in the PDB with an *e*-value > 10^−5^ or a sequence identity to the yeast protein of interest <30%. Partial matches occur when at least two predicted metal-binding residues overlapped properly in the structural comparison, as opposed to complete matches, which occurred when all predicted metal-binding residues overlapped correctly.

**Figure 2.**
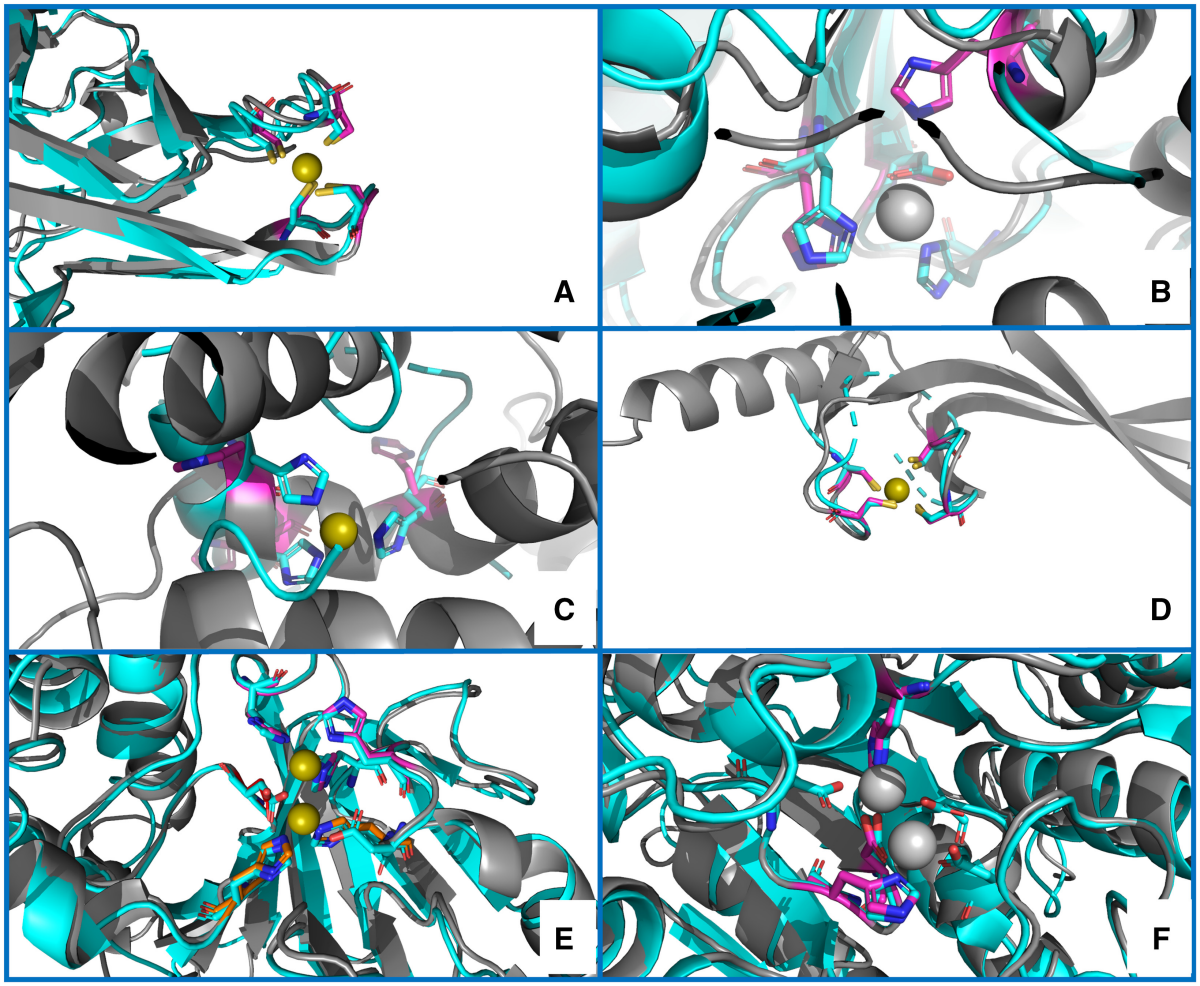
Examples of structure predictions by MOM. (A) a correct prediction, validated by superimposition to a distant homolog structure [PDB ID 5ZLQ (Furukawa *et al.* 2018)]; (B) a partial match, with two out of three residues correctly superimposed to the metal ligands of the manganese(II) ion of a distant homolog structure [PDB ID 5M45 (Mus *et al.* 2017)]; (C) an inaccurate prediction [superimposed to the 1BM6_2 (Li *et al.* 1998) MetalPDB site], in which two of the three predicted His have a plausible spatial disposition, but the third His cannot be regarded a putative ligand since its positioning in the α-helix prevents any movement to form an MBS in the presence of the metal ion; (D) a correct prediction for a protein lacking a homolog with known structure, validated by superposition of the AlphaFold structural model to the closest MetalPDB site [3BVO_1 (Bitto *et al.* 2008)] identified by MoM; (E) a correct prediction for two zinc(II) sites in spatial proximity, validated by superimposition to a homolog structure [PDB ID 2P18 (Sousa Silva *et al.* 2008)], which contains a dinuclear zinc(II) cluster; (F) a partial match, where MoM predicted only one site containing a subset of the ligands to the two manganese(II) ions present in a homolog structure (1WVB). The color code is as follows: gray, AlphaFold structural models; fuchsia, predicted ligand residues; cyan, homolog structures or closest MetalPDB site. The zinc(II) ions are shown as olive green spheres, whereas all other metal ions are shown as grey spheres

We then inspected the 16 predicted zinc(II) proteins whose experimentally characterized homologs bear different metals. We obtained satisfactory superimpositions with the experimental sites for nine proteins (56%), as shown in Fig. 2E and F. For example (Fig. 2E), for the protein with UniProt ID Q05584 (cytoplasmic hydroxyacylglutathione hydrolase), the two sites predicted by our tool overlapped almost perfectly with all the metal-binding residues observed in the experimental structures of various homologs. Notably, the protein is annotated as a zinc(II) enzyme in UniProt. The proteins having a zinc(II)-binding homolog were separated in two groups, depending on their sequence similarity. The superimposition verified the positions predicted by our method for eight out of eight proteins (100%) that had only distant homologs (Table 2 and Fig. 2A and B). The predicted sites were exactly overlaid to the real ones in 32 out of the 46 (70%) near-homolog structures containing zinc(II), whereas in another seven structures (15%), our prediction only partially matched the experimental MBS. Finally, our prediction did not overlap with the experimentally observed MBS in seven cases (15%).

The 31 models with no structural information from homologs are of high interest since they may contain zinc(II)-binding sites never observed before. The reliability of the predicted sites was qualitatively evaluated by the superimposition to the site used for the prediction. For 11 structures (35%), the overlap was perfect (Fig. 2D), whereas the outcomes for 13 proteins (42%) were deemed satisfactory, given that the residues are arranged in a way that appears compatible with the binding geometry observed in the reference site. On the other hand, for seven proteins (23%), the prediction appeared unreliable, e.g. because the putative ligands were in secondary structure elements preventing the reorientation of their SCs to coordinate the metal (Fig. 2C).

Overall, MoM demonstrated a satisfactory performance in a real life scenario, namely the analysis of the proteome of an entire organism. Cumulatively, with a *d*_min threshold of 0.35 Å, MoM had an error rate (false discovery rate, FDR, given by the ratio of FP over the total number of positive predictions) for all its predicted MBSs of 24% and a precision of 76% (Fig. 1). To obtain further insight into the performance of MoM, we performed a comparison with a previously published dataset consisting of 229 zinc(II)-proteins identified by sequence-based bioinformatics prediction of the MBS and detected by mass spectrometry in zinc-replete cells (Wang *et al.* 2018). Out of these 229 proteins, we analysed 157 structural models of sufficient quality. With a *d*_min threshold of 0.35, MoM predicted the existence of a zinc(II) MBS for 151 of these models (96% of the dataset), yielding a FN rate of 4%. At the level of individual residues, in 130 proteins (86% of the dataset), the current prediction included all or all but one of the previously proposed ligands.

## 4 Discussion

In this work we developed a tool for the prediction of zinc(II) MBSs in the 3D structure of proteins, by combining an NN and a post-processing geometry filter. By design, the minimum number of metal ligands in the site is three, implying that the tool is most suited for the prediction of intra-chain sites (Tran and Krężel 2021). The role of the NN is to extract from the input 3D structure all the groups of residues of the CHED type (Cys, His, Asp, Glu) (Babor *et al.* 2008) that constitute potential binding sites. We trained the NN on an extensive dataset of physiological zinc(II) MBSs. For the validation process, we clustered homologous proteins so that their sites would not be present in the training and validation sets at the same time; 20% of the experimental MBSs were kept as the independent validation dataset. Each of the candidate sites identified by the NN in the input 3D structure is filtered for geometrical similarity to all known MBSs with the same metal-binding pattern, by comparing their distance matrices for the Cα and Cβ atoms. By fixing a threshold for the deviation between the matrices (*d*_min), FP sites are significantly reduced. The application of this similarity filter is justified by our previous work demonstrating that for zinc(II) (Andreini *et al.* 2011) as well as iron-sulfur proteins (Andreini *et al.* 2009), the same metal-binding motifs can occur in completely different folds. In fact, the MBSs of about 77% of all zinc(II)-protein superfamilies can be grouped in just 10 clusters (Andreini *et al.* 2011). In a similar fashion, related metal-binding structural motifs (which are similar to the MBS concept used here) can be identified within different, evolutionarily distant protein structures (Bromberg *et al.* 2022).

The recall of our predictor was nearly 92% with a *d*_min value of 0.35 Å. We applied the trained predictor to a dataset of apo-sites [i.e. sites extracted from the 3D structure of zinc(II)-proteins experimentally determined in the absence of their metal cofactor]. For this group of structures, there is no preorganization of the protein residues surrounding the metal ion, as it is instead the case for MBSs taken from holo structures after removing the ion from the coordinate file. Indeed, we observed a lower recall, of about 79%, which is still quite satisfactory. Notably, it is known that backbone rearrangements are typically modest upon metalation of apo-sites (Babor *et al.* 2005, Bazayeva *et al.* 2023), which we exploited in the design of MoM as well as of other related tools (Nguyen and Kleingardner 2021, Sánchez-Aparicio *et al.* 2021). To obtain an indication of the FPR of the predictor, we examined 500 structures of proteins with no reported interaction with metal ions of physiological relevance. MoM proposed the presence of an MBS in 14% of them.

Our method may be used to predict all the zinc(II) proteins of a given organism starting from its proteome sequence. To demonstrate this, we investigated the proteome of the yeast *S.cerevisiae*. We decided to focus on this organism also because of the availability of a combined bioinformatics and proteomics analysis that provided a list of yeast zinc(II) proteins with experimental validation (Wang *et al.* 2018). Our tool predicted the occurrence of a zinc(II) MBS in 191 proteins out of 1500 analysed, of which 77 had a deposited experimental structure. This allowed us to calculate an independent estimate of the precision (i.e. the percentage of predicted sites that are actually correct), namely 80.5%. Further validation of MoM resulted from the analysis of the remaining 114 predicted yeast zinc proteins against the experimental structures of homologous proteins from other organisms; the precision in this subgroup of proteins was 72%. By combining the two datasets, we obtain an overall estimate of the precision of the MBS predictions for the yeast proteome at about 76% and a false discovery rate of 24%. Finally, for a previously reported list of yeast zinc(II) proteins obtained by a combination of bioinformatics methods and mass spectrometry, we had a recall of 86%. These results are in between the recall measured for holo- and apo-sites at the 0.35 Å threshold that we used here.

Our tool can be compared with other software that perform the same task, developed or updated in the past few years. In particular, a deep-learning approach recently has been implemented in Metal3D (Dürr *et al.* 2023). With a value of the *p* parameter of Metal3D equal to 0.75, the latter tool achieves a recall close to 80% and a precision of about 82% for sites containing at least three ligands. The recall and precision of Metal3D have been estimated only on crystallographic structures of the holo form of zinc(II) proteins, hence only for sites already in the metal-bound conformation. We thus checked whether the structural rearrangements possibly occurring upon metalation reduced the software performance of Metal3D by using apo-structures as input, without finding any compelling evidence for such a trend. However, we noted that Metal3D seems more sensitive than our method to incomplete structures or to changes in the rotameric state of the metal ligands between the apo- and holo-structure, possibly because for such inputs the voxelized site computed by Metal3D is not a correct representation of the holo-MBS. An intriguing example is that of PDB entry 1T38 (Daniels *et al.* 2004), whose zinc(II) site is unoccupied due to the additional tag present in the construct (Daniels *et al.* 2004), leading to a significant rotation of the SC of His29 as compared with the corresponding holo- structure [PDB code 1YFH (Duguid *et al.* 2005)]; in addition, the most N-terminal ligand, Cys5, is not observed in 1T38. Our tool but not Metal3D could identify the site in the 1T38 apo-structure. However, the AlphaFold model of the protein structure contained a properly preorganized apo-site, which Metal3D could detect with very high confidence. MoM featured a recall of about 90% on crystallographic structures of holo-zinc(II) proteins and of about 83% for the corresponding apo-structures, whereas the analysis of the predictions for the AlphaFold models of all yeast proteins indicated a recall in the range 75%–85%, depending on the chosen reference dataset, and precision of around 76%. We can thus conclude that our tool has a performance practically aligned with that of Metal3D despite its simpler architecture. Its simplicity allows the present method to achieve comparatively faster calculations, enabling the analysis of a full proteome, such as yeast, in a matter of hours.

Other related tools are BioMetAll (Sánchez-Aparicio *et al.* 2021) and MIB2 (Lu *et al.* 2022). Besides their different methodologies, these tools are not appropriate for high-throughput applications to entire proteomes. MIB2 is available only as a web server designed for testing individual structures, whereas BioMetAll outputs for each input structure multiple possible sites, with no quantitative ranking of the predictions. AlphaFill instead fills the apo-sites in the AlphaFold models by docking the ions present in homologous proteins with a deposited PDB structure (Hekkelman *et al.* 2023), thus relying strictly on the detection of a homology relationship.

In summary, we developed the MoM tool for the identification of potential zinc(II) MBSs in 3D structural models. MoM can be conveniently run on entire proteomes, in order to obtain a prediction of any organism’s entire zinc(II) proteome. The tool is available at https://github.com/cerm-cirmmp/Master-of-metals. MoM has been applied to the yeast proteome, and the predictions validated against different datasets. Besides the precision, which we discussed in the previous paragraphs, our approach featured a false discovery rate of 24% with a threshold of 0.35 Å, which corresponds to three predicted MBSs in four being correct. When necessary, this aspect can be improved by applying a more stringent filter: using a threshold of 0.30 Å reduces the recall by less than one tenth while reducing FP by about one-third (Table 1). In any case, visual inspection of the results is strongly recommended.

## Supplementary Material

btad653_Supplementary_Data

## Data Availability

All data used for training and validating MoM is available at https://github.com/cerm-cirmmp/Master-of-metals. The results of the yeast proteome analysis are available in the online supplementary material.

## References

[btad653-B1] Andreini C , Bertini I, Cavallaro G. Minimal functional sites allow a classification of zinc sites in proteins. PLoS One 2011;6:e26325. 22043316 10.1371/journal.pone.0026325 PMC3197139

[btad653-B2] Andreini C , Bertini I, Cavallaro G et al Structural analysis of metal sites in proteins: non-heme iron sites as a case study. J Mol Biol 2009;388:356–80. 19265704 10.1016/j.jmb.2009.02.052

[btad653-B3] Andreini C , Bertini I, Rosato A. Metalloproteomes: a bioinformatic approach. Acc Chem Res 2009;42:1471–9. 19697929 10.1021/ar900015x

[btad653-B4] Andreini C , Cavallaro G, Lorenzini S. FindGeo: a tool for determining metal coordination geometry. Bioinformatics 2012;28:1658–60. 22556364 10.1093/bioinformatics/bts246

[btad653-B5] Andreini C , Cavallaro G, Lorenzini S et al MetalPDB: a database of metal sites in biological macromolecular structures. Nucleic Acids Res 2013;41:D312–9. 23155064 10.1093/nar/gks1063 PMC3531106

[btad653-B6] Andreini C , Rosato A. Structural bioinformatics and deep learning of metalloproteins: recent advances and applications. Int J Mol Sci 2022;23:7684. 35887033 10.3390/ijms23147684 PMC9323969

[btad653-B7] Babor M , Gerzon S, Raveh B et al Prediction of transition metal-binding sites from apo protein structures. Proteins Struct Funct Bioinf 2008;70:208–17. 10.1002/prot.21587 17657805

[btad653-B8] Babor M , Greenblatt HM, Edelman M et al Flexibility of metal binding sites in proteins on a database scale. Proteins 2005;59:221–30. 15726624 10.1002/prot.20431

[btad653-B9] Barnett JP , Scanlan DJ, Blindauer CA. Protein fractionation and detection for metalloproteomics: challenges and approaches. Anal Bioanal Chem 2012;402:3311–22. 22302168 10.1007/s00216-012-5743-y

[btad653-B10] Bazayeva M , Laveglia V, Andreini C et al Metal-induced structural variability of mononuclear metal-binding sites from a database perspective. J Inorg Biochem 2023;238:112025. 36270040 10.1016/j.jinorgbio.2022.112025

[btad653-B11] Bitto E , Bingman CA, Bittova L et al Structure of human J-type Co-chaperone HscB reveals a tetracysteine metal-binding domain. J Biol Chem 2008;283:30184–92. 18713742 10.1074/jbc.M804746200 PMC2573069

[btad653-B12] Bromberg Y , Aptekmann AA, Mahlich Y et al Quantifying structural relationships of metal-binding sites suggests origins of biological electron transfer. Sci Adv 2022;8:eabj3984. 35030025 10.1126/sciadv.abj3984 PMC8759750

[btad653-B13] Chen AY , Adamek RN, Dick BL et al Targeting metalloenzymes for therapeutic intervention. Chem Rev 2019;119:1323–455. 30192523 10.1021/acs.chemrev.8b00201 PMC6405328

[btad653-B14] Chen Z , Wang Y, Zhai Y-F et al ZincExplorer: an accurate hybrid method to improve the prediction of zinc-binding sites from protein sequences. Mol Biosyst 2013;9:2213–22. 23861030 10.1039/c3mb70100j

[btad653-B15] Daniels DS , Woo TT, Luu KX et al DNA binding and nucleotide flipping by the human DNA repair protein AGT. Nat Struct Mol Biol 2004;11:714–20. 15221026 10.1038/nsmb791

[btad653-B16] Degtyarenko K. Bioinorganic motifs: towards functional classification of metalloproteins. Bioinformatics 2000;16:851–64. 11120676 10.1093/bioinformatics/16.10.851

[btad653-B17] Duguid EM , Rice PA, He C. The structure of the human AGT protein bound to DNA and its implications for damage detection. J Mol Biol 2005;350:657–66. 15964013 10.1016/j.jmb.2005.05.028

[btad653-B18] Dürr SL , Levy A, Rothlisberger U. Metal3D: a general deep learning framework for accurate metal ion location prediction in proteins. Nat Commun 2023;14:2713. 37169763 10.1038/s41467-023-37870-6 PMC10175565

[btad653-B19] Feehan R , Franklin MW, Slusky JSG. Machine learning differentiates enzymatic and non-enzymatic metals in proteins. Nat Commun 2021;12:3712. 34140507 10.1038/s41467-021-24070-3 PMC8211803

[btad653-B20] Furukawa Y , Lim C, Tosha T et al Identification of a novel zinc-binding protein, C1orf123, as an interactor with a heavy metal-associated domain. PLoS One 2018;13:e0204355. 30260988 10.1371/journal.pone.0204355 PMC6160046

[btad653-B21] Gladyshev VN , Zhang Y. Comparative genomics analysis of the metallomes. Met Ions Life Sci 2013;12:529–80. 23595683 10.1007/978-94-007-5561-1_16

[btad653-B22] Grime GW , Zeldin OB, Snell ME et al High-throughput PIXE as an essential quantitative assay for accurate metalloprotein structural analysis: development and application. J Am Chem Soc 2020;142:185–97. 31794207 10.1021/jacs.9b09186

[btad653-B23] Hekkelman ML , De Vries I, Joosten RP et al AlphaFill: enriching alphafold models with ligands and cofactors. Nat Methods 2023;20:205–13. 36424442 10.1038/s41592-022-01685-y PMC9911346

[btad653-B24] Hennigar SR , McClung JP. Nutritional immunity: starving pathogens of trace minerals. Am J Lifestyle Med 2016;10:170–3. 30202269 10.1177/1559827616629117 PMC6124953

[btad653-B25] Hu X , Dong Q, Yang J et al Recognizing metal and acid radical ion-binding sites by integrating ab initio modeling with template-based transferals. Bioinformatics 2016;32:3260–9. 27378301 10.1093/bioinformatics/btw396 PMC5079472

[btad653-B26] Huang Y , Niu B, Gao Y et al CD-HIT suite: a web server for clustering and comparing biological sequences. Bioinformatics 2010;26:680–2. 20053844 10.1093/bioinformatics/btq003 PMC2828112

[btad653-B27] Ireland SM , Martin ACR. Zincbindpredict—prediction of zinc binding sites in proteins. Molecules 2021;26:26. 10.3390/molecules26040966 PMC7918553 33673040

[btad653-B28] Jumper J , Evans R, Pritzel A et al Highly accurate protein structure prediction with AlphaFold. Nature2021a;596:583–9. 34265844 10.1038/s41586-021-03819-2 PMC8371605

[btad653-B29] Jumper J , Evans R, Pritzel A et al Applying and improving AlphaFold at CASP14. Proteins Struct Funct Bioinf2021b;89:1711–21. 10.1002/prot.26257 PMC9299164 34599769

[btad653-B30] Koohi-Moghadam M , Wang H, Wang Y et al Predicting disease-associated mutation of metal-binding sites in proteins using a deep learning approach. Nat Mach Intel 2019;1:561–7.

[btad653-B31] Laveglia V , Giachetti A, Sala D et al Learning to identify physiological and adventitious metal-binding sites in the three-dimensional structures of proteins by following the hints of a deep neural network. J Chem Inf Model 2022;62:2951–60. 35679182 10.1021/acs.jcim.2c00522 PMC9241070

[btad653-B32] Li YC , Zhang X, Melton R et al Solution structure of the catalytic domain of human stromelysin-1 complexed to a potent, nonpeptidic inhibitor. Biochemistry 1998;37:14048–56. 9760240 10.1021/bi981328w

[btad653-B33] Lin Y-F , Cheng C-W, Shih C-S et al MIB: metal ion-binding site prediction and docking server. J Chem Inf Model 2016;56:2287–91. 27976886 10.1021/acs.jcim.6b00407

[btad653-B34] Lu C-H , Chen C-C, Yu C-S et al MIB2: metal ion-binding site prediction and modeling server. Bioinformatics 2022;38:4428–9. 35904542 10.1093/bioinformatics/btac534

[btad653-B35] Mertz W. Review of the scientific basis for establishing the essentiality of trace elements. Biol Trace Elem Res 1998;66:185–91. 10050919 10.1007/BF02783137

[btad653-B36] Mus F , Eilers BJ, Alleman AB et al Structural basis for the mechanism of ATP-dependent acetone carboxylation. Sci Rep 2017;7:7234. 28775283 10.1038/s41598-017-06973-8 PMC5543143

[btad653-B37] Nguyen H , Kleingardner J. Identifying metal binding amino acids based on backbone geometries as a tool for metalloprotein engineering. Protein Sci 2021;30:1247–57. 33829594 10.1002/pro.4074 PMC8138524

[btad653-B38] Nordberg M , Nordberg GF. Trace element research-historical and future aspects. J Trace Elem Med Biol 2016;38:46–52. 27238729 10.1016/j.jtemb.2016.04.006

[btad653-B39] Passerini A , Andreini C, Menchetti S et al Predicting zinc binding at the proteome level. BMC Bioinformatics 2007;8:39. 17280606 10.1186/1471-2105-8-39 PMC1800866

[btad653-B40] Putignano V , Rosato A, Banci L et al MetalPDB in 2018: a database of metal sites in biological macromolecular structures. Nucleic Acids Res. 2018;46:D459–64. 29077942 10.1093/nar/gkx989 PMC5753354

[btad653-B41] Sánchez-Aparicio J-E , Tiessler-Sala L, Velasco-Carneros L et al BioMetAll: identifying metal-binding sites in proteins from backbone preorganization. J Chem Inf Model 2021;61:311–23. 33337144 10.1021/acs.jcim.0c00827

[btad653-B42] Senior AW , Evans R, Jumper J et al Improved protein structure prediction using potentials from deep learning. Nature 2020;577:706–10. 31942072 10.1038/s41586-019-1923-7

[btad653-B43] Shi W , Chance MR. Metalloproteomics: forward and reverse approaches in metalloprotein structural and functional characterization. Curr Opin Chem Biol 2011;15:144–8. 21130021 10.1016/j.cbpa.2010.11.004 PMC3040278

[btad653-B44] Silva MS , Barata L, Ferreira AEN et al Catalysis and structural properties of *Leishmania infantum* glyoxalase II: trypanothione specificity and phylogeny. Biochemistry 2008;47:195–204. 18052346 10.1021/bi700989m

[btad653-B45] Tran JB , Krężel A. InterMetalDB: a database and browser of intermolecular metal binding sites in macromolecules with structural information. J Proteome Res 2021;20:1889–901. 33502860 10.1021/acs.jproteome.0c00906 PMC8023803

[btad653-B46] Varadi M , Anyango S, Deshpande M et al AlphaFold protein structure database: massively expanding the structural coverage of protein-sequence space with high-accuracy models. Nucleic Acids Res 2022;50:D439–44. 34791371 10.1093/nar/gkab1061 PMC8728224

[btad653-B47] Wang Y , Weisenhorn E, MacDiarmid CW et al The cellular economy of the *Saccharomyces cerevisiae* zinc proteome. Metallomics 2018;10:1755–76. 30358795 10.1039/c8mt00269j PMC6291366

[btad653-B48] wwPDB Consortium. Protein Data Bank: the single global archive for 3D macromolecular structure data. Nucleic Acids Res. 2019;47:D520–28. 30357364 10.1093/nar/gky949 PMC6324056

[btad653-B49] Zhang Y , Zheng J. Bioinformatics of metalloproteins and metalloproteomes. Molecules 2020;25:3366. 32722260 10.3390/molecules25153366 PMC7435645

[btad653-B50] Zheng H , Cooper DR, Porebski PJ et al CheckMyMetal: a macromolecular metal-binding validation tool. Acta Crystallogr D Struct Biol 2017;73:223–33. 28291757 10.1107/S2059798317001061 PMC5349434

