# Supplementary material for: Hunting down zinc(II)-binding sites in proteins with distance matrices
Source: Bioinformatics. 2023 Oct 25;39(11):btad653. doi: 10.1093/bioinformatics/btad653 (PMC10630175; doi:10.1093/bioinformatics/btad653)
Supplement: btad653_Supplementary_Data [file btad653_supplementary_data.zip › TableS1r1.docx]

**Hunting down zinc(II)-binding sites in proteins with distance matrices**

Vincenzo Laveglia^1^, Milana Bazayeva^1,2^, Claudia Andreini^1,2,3^, Antonio Rosato^1,2,3,*^

1 Department of Chemistry, University of Florence, Via della Lastruccia 3, 50019 Sesto Fiorentino, Italy.

2 Magnetic Resonance Center (CERM), University of Florence, Via Luigi Sacconi 6, 50019 Sesto Fiorentino, Italy

3 Consorzio Interuniversitario di Risonanze Magnetiche di Metallo Proteine, Via Luigi Sacconi 6, 50019 Sesto Fiorentino, Italy.

**Supplementary Materials**

| **Pattern** | **# of sites** | **% of total** | **Geometries** |
| --- | --- | --- | --- |
| CYC CYS CYS CYS | 5646 | 25.58 % | Tetrahedron (regular) 69.53%  Tetrahedron (distorted) 12.81% |
| HIS HIS HIS | 1674 | 7.58 % | Irregular (n/a) 41.89%  Tetrahedron (regular) 33.10% |
| CYS CYS HIS CYS | 1511 | 6.85% | Tetrahedron (regular) 81.73%  Tetrahedron (distorted) 9.99% |
| CYS HIS CYS CYS | 1363 | 6.17 % | Tetrahedron (regular) 85.33%  Tetrahedron (distorted) 8.22% |
| CYS CYS CYS HIS | 1005 | 4.55% | Tetrahedron (regular) 68.96%  Tetrahedron (distorted) 13.63% |
| CYS CYS HIS HIS | 992 | 4.49 % | Tetrahedron (regular) 73.29%  Tetrahedron (distorted) 14.26% |
| HIS HIS GLU | 787 | 3.57 % | Irregular (n/a 70.54%  Octahedron (distorted) 6.83% |
| CYS CYS CYS | 740 | 3.35 % | Irregular (n/a) 25.53%  Tetrahedron (regular) 24.87% |
| HIS HIS ASP ASP | 522 | 2.36 % | Octahedron (regular) 52.80%  Octahedron (distorted) 13.20% |
| HIS HIS HIS ASP | 469 | 2.12 % | Irregular (n/a) 58.21%  Trigonal bipyramid (regular) 12.37% |
| CYS HIS CYS | 407 | 1.84% | Tetrahedron (regular) 39.61%  Tetrahedron (distorted) 28.36% |
| HIS ASP HIS HIS | 389 | 1.76 % | Tetrahedron (regular) 70.95%  Tetrahedron (distorted) 12.60% |

**Table S1. The 12 most common metal-binding patterns observed in the holo structures of mononuclear zinc(II) proteins extracted from MetalPDB.** Geometries are taken from MetalPDB and have been computed taking into account also the presence of exogenous ligands (e.g. water. anions); only the two most common geometries are shown for each group of patterns.

**Table S2. List of the 191 predicted zinc-proteins, identified by their UniProt code, with a quality flag (manually assigned).** For proteins with an experimental structure available, the quality flag is CORRECT/WRONG, depending on whether the predicted site was indeed in the experimental structure. For all other proteins, the quality flag corresponds to the labeling in Figure 1, with a further separation of positive sites into perfectly (CORRECT) or partly (PARTIAL MATCH) overlapping; in the latter group a subset of all experimental zinc(II) ligands was predicted by MoM. Table S2 is available as a csv file.
